# A systematic review of prognosis of ABO blood group and rhesus factor on outcomes in patients with bladder cancer

**DOI:** 10.1097/MD.0000000000030893

**Published:** 2022-09-30

**Authors:** Haiming Yang, Jingxin Yan

**Affiliations:** a Department of Interventional Therapy, Qinghai University, Xining, China; b Department of Postgraduate, Qinghai University, Xining, China.

**Keywords:** ABO blood group, bladder cancer, prognosis, rhesus factor, systematic review

## Abstract

Bladder cancer (BC) is the 10th most common malignancy worldwide, and some studies reported that ABO blood type or/and rhesus factor has been identified as a prognostic oncologic marker for patients with BC. We carried out a systematic review to assess the prognosis of ABO blood group and rhesus factor on outcomes in patients with bladder cancer.

**Methods:**

We searched databases through February 2022 for studies assessing blood group on outcomes in patients with bladder cancer.

**Results:**

We included ten studies with 15,204 participants. We found that blood type A is relevant to non-muscle-invasive BC patients treated with transurethral resection of bladder tumor and blood type B patients have a lower incidence of disease recurrence and progression. Blood type O and non-O blood type have not been found to be related to disease recurrence. However, in multivariable analyses, blood type O and non-O blood type are associated with cancer-specific mortality (CSM). Other than that, blood type B doesn’t have statistical significance for BC patients accepted radical cystectomy (RC). The same results showed in blood type AB non-muscle-invasive bladder cancer patients treated with RC.

**Conclusions:**

Our study confirmed that a particular association of blood type for prognosis of patients with BC, and ABO blood group antigen expression can be suitable biomarkers for BC. We also found that rhesus factor has no impact on prognosis of BC patients.

## 1. Introduction

Bladder cancer (BC) is the 10th most common malignancy worldwide, with an estimated 549,000 new cases and 200,000 deaths in 2018 and projections for these to reach more than 670,000 by 2025.^[1]^ Other than certain occupational exposures to chemical and water contaminants, cigarette smoking is the main risk factor for BC.^[2]^ Approximately one-fourth of patients with BC present with muscle-invasive disease while the rest of those with non-muscle-invasive bladder cancer (NMIBC) at diagnosis, which may progress to muscle invasion.^[3]^ Transurethtal resection of bladder tumor (TURBT) and radical cystectomy (RC) with lymphadenectomy are recommended for NMIBC^[4]^ and high-grade bladder urothelial cancer such as BCG-resistant carcinoma in situ (CIS), respectively.^[5]^ The survival period of the majority of patients with advanced BC are less than 2 years, and the prognosis goes even worse once BC recurs after surgery.^[6]^ Improving risk stratification before surgery represents a priority to enhancing patient outcomes. Some studies reported that the serum metabolites, proteins, or inflammatory cells can be used for BC detection. Besides, in the view of BC having different prognoses and responses to treatment as a reflection of molecular heterogeneity, blood serum a novelty approach as the metabolomic for the identification of biomarkers in BC.^[7]^ There are potential biomarkers such as blood type and other molecular biology, as it may reflect underlying cancer biology.^[8]^ In addition, biomarkers may improve the ability of predictive models^[9]^ to patients for treatment, such as perioperative chemotherapy and altered surveillance protocols.

The ABO gene is located on chromosome for specific glycosyltransferases which is finally converted into A or B antigen.^[10–12]^ O gene encodes a nonfunctional glycosyltransferase and O blood group was associated with the absence of lymph node metastasis in urinary carcinoma patients.^[13,14]^ Based on those studies, the ABO blood type and rhesus factor has emerged as an inexpensive, readily available markers that is associated with prognosis of patients with various malignancies including bladder, renal and pancreatic cancer.^[13–16]^

Some studies reported that ABO blood type or/and rhesus factor has been identified as a prognostic oncologic marker for patients with BC, which prompted us to conceive a safe and inexpensive method to determine the risk of recurrence and mortality. However, there are substantial inconsistencies between reports. Although some conclusions have been made, the impact of the ABO and the rhesus blood group system on outcomes has not been systematically reviewed. Therefore, in the current study, we performed a systematic review to assess the ABO blood type and rhesus factor on prognosis of BC patients who underwent surgery.

## 2. Methods

### 2.1. Search strategy and trial selection

This systematic review was performed according to the Preferred Reporting Items for Systematic Reviews and Meta-Analyses (PRISMA) guidelines.^[17]^ Two researchers independently searched the Pubmed, Embase, Cochrane liberary, CNKI and Wanfang database from 2000 to February 2022. The mesh and keywords used for the searches included: “ABO blood type”, “rhesus blood group”, “bladder cancer”, “urothelial carcinoma of the bladder” and “prognosis”.

### 2.2. Study inclusion criteria

The trails included in this study must meet the criteria: original research reported the prognosis of patients with BC and known ABO blood type; a retrospective study design or randomized controlled trails (RCTs); with a total score of Newcastle–Ottawa scale (NOS) >5; and availability of the full-text article. Exclusion was as follows: valid data were unavailable or data not completed or inaccurate so that the study could not be analyzed; relevant outcome indexes were not reported; case reports, comments, meta-analysis, systematic review, reviews, abstracts, editorials, and thesis; and duplicate publications.

### 2.3. Data extraction and quality assessment

The following data from the included studies were extracted a by 2 researchers: for each study, author, year of publication, sample size, mean age, intervention measure, type of procedure, and any outcome that met the inclusion criteria. The quality of our included trails were assessed and scored by two researchers and checked by the third researcher using the NOS.^[18]^

## 3. Results

### 3.1. Studies inclusion

**Figure 1.**
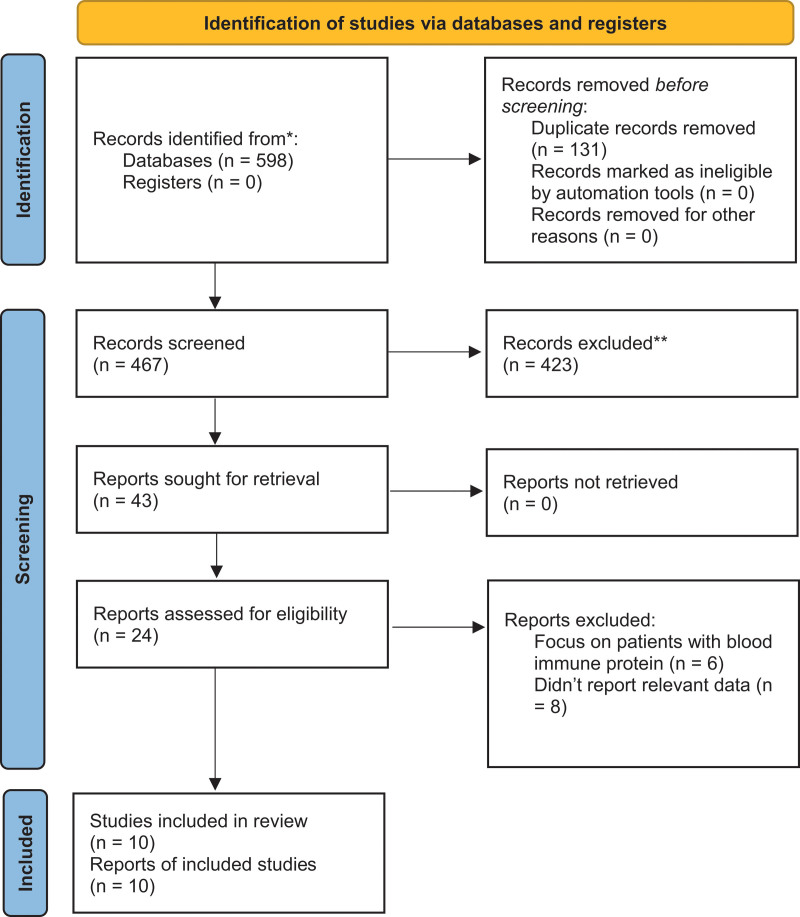
Selection algorithm and numbers of included and excluded studies.

Initial database searches identified 598 publications. After removing 86 duplicated articles, we further excluded 467 articles by screening their titles and abstracts. Following review of the full-text articles for the remaining 24 publications, we identified 15 publications reporting potentially relevant studies. Consequently, we included 3 retrospective studies^[19–21]^ published in Chinese and 7 retrospective studies^[22–28]^ published in English in this systematic review. Figure 1 displays the selection algorithm and numbers of included and excluded studies. All data were derived from previously published studies, so the ethical approval is not required.

### 3.2. Study characteristics, patient populations and study quality

The included articles were published from 2014 to 2019. The sample size varied from 103 to 7906, reaching a total of 15,204 participants. All included studies reported the ABO blood type of participants, and 10 included studies reported the treatment of participants and 4 studies^[22,24,26,27]^ reported the data on Rhesus blood group system. The main study characteristics are shown in Table 1.

**Table 1:** Characteristics of studies included in the systematic review.

Study	Number of patients(O/A/B/AB)	Rhesus (+)/Rhesus(-)	Men/women	Age (years)	Histopathology	Surgery procedure
Engel O. 2015	189/216/73/33	414/97	401/110	Median (IQR): 66 (49–73)	muscle-invasive disease or recurrent Ta, T1 or refractory CIS	RC and chemotherapy
Gershman B. 2014	913/881/216/76	NA	1712/374	Median (IQR): 68 (62–74)	NA	RC and chemotherapy
Moschini M. 2016	341/277/83/27	630/98	600/128	Mean:67.4 Median(IQR): 68 (61–74)	NA	RC and extended pelvic lymph node dissection
Su¨er E. 2014	110/180^a^	247/43	260/30	NA	NA	RC, pelvic lymphadenectomy and urinary diversion
D’ANDREA D. 2017	185/190/46/27	383/65	373/75	Median(IQR): 65 (60–71)	NA	RC and lymphadenectomy
Klatte T 2014	3738/2748/888/532	NA	6290/1616	NA	NA	RC with bilateral pelvic lymphadenectomy
Wang L. 2016	29/40/22/12	NA	78/25	Mean(range): 65.85(35‐86)	MIBC	RC with bilateral pelvic lymph node dissection
He L.L. 2017	178/241/66/36	NA	395/126	Male:55~96 Female:61~89	Primary NMIBC	TURBT and chemotherapy
Wang X.H. 2019	628/505/679/233	NA	1579/466	Median(IQR):63(58‐73)	NMIBC	TURBT and chemotherapy
Cheng L. 2019	114/130/107/37	NA	NA	NA	NMIBC	TURBT and chemotherapy

a: in this study, a total of 180 patients were non-O, while the 110 patients had the blood group O (NMIBC = non-muscle-invasive bladder cancer, TURBT =transurethral resection of bladder tumor, RC = radical cystectomy, CIS = carcinoma in situ, NA = not applicable, MIBC = muscle-invasive bladder cancer).

The included observational studies met most of the quality assessment criteria, and all these studies were scaled as a total score of >5, indicating a low risk of bias. The details are shown in Table 2.

**Table 2:** Results of quality assessment using Newcastle–Ottawa scale for cohort studies.

Study selection	Representativeness of the exposed cohort	Selection of the nonexposed cohort	Ascertainment of exposure	Demonstration that outcome of interest was not present at start of study	Comparability of cohorts on the basis of the design or analysis	Assessment of outcome	Follow-up long enough for outcomes to occur	Adequacy of follow-up of cohorts	Quality score
Su¨er E.	0	0	1	1	2	1	1	1	7
Wang,X.H.	1	1	0	1	2	1	1	1	8
Gershman B.	1	1	1	1	2	1	1	1	9
Engle O.	1	1	1	1	2	1	1	1	9
Cheng,L.	0	1	1	1	1	1	1	1	7
Gershman M.	0	0	1	1	2	1	1	1	7
D’ANDREA D.	1	1	1	1	2	1	1	1	9
Klatte T.	1	1	1	1	2	1	1	1	9
Wang, L.	1	0	1	1	2	1	1	1	8
He, L.L.	1	1	1	1	1	0	1	1	7

### 3.3. Results regarding blood type non-O

Based on 4 studies^[22,25–27]^ reported BC patients treated with RC and measured the blood type non-O (ref: O) on prognosis of included patients, the outcomes of pooled results did not demonstrate an influence of non-O blood group on disease recurrence-free survival (RFS), cancer-specific survival (CSS) and overall survival (OS) of the patients treated with RC for BC. What’s more, the blood type non-O has no association with an advanced pathologic tumor stage and lymph node metastasis in according to Engle’s study.^[26]^ Gershman et al^[25]^ reported that disease recurrence (HR: 1.18, 95% CI: 1.01‐1.37) and cancer-specific mortality (CSM) (HR: 1.20, 95% CI: 1.04‐1.39) can be weakly associated with blood type O and non-O from univariable analyses. Interestingly, no association were found in disease recurrence (HR: 1.18, 95% CI: 0.99‐1.39) while CSM (HR: 1.22, 95% CI: 1.04‐1.44) was still associated with blood type O and non-O from multivariable analyses.

### 3.4. Results regarding blood type A

Although all included studies blood type A (Ref: O) on prognosis of included patients, there remain controversy. For BC patients treated with RC, based on 6 studies, only Gershman et al.^[25]^ reported that blood type A can be weakly associated with disease recurrence (HR: 1.18, 95% CI: 1.01‐1.37) and CSM (HR: 1.23, 95% CI: 1.06‐1.44). The other studies did not demonstrate an influence of blood group A on disease RFS, CSS and OS of the patients treated with RC.

In patients treated with TURBT, based on 3 studies^[19–21]^ from China, blood type A (Ref: O) can be associated with the prognosis of patients with TURBT for NMIBC. However, there remains controversial conclusion among studies. Cheng et al^[19]^ reported that patients with blood type O have higher recurrence and progression rate than type A while Wang et al^[21]^ and He et al^[20]^ reported that patients with blood type O have lower recurrence and progression rate than type A.

### 3.5. Results regarding blood type B

Compared with blood type O, blood type B has no statistical association with CSM, disease recurrence, overall mortality, OS, CSS and RFS. What’s more, 1 study^[26]^ reported that blood type B is not a risk predictor on advanced pathologic tumor stage or positive lymph node metastasis.

However, for NMIBC patients treated with TURBT, based on Wang^[21]^ study and Cheng^[19]^ study, blood type B indicate a lower incidence of disease recurrence and progression.

### 3.6. Results regarding blood type AB

Compared with blood type O, blood type AB has no statistical association with prognosis of BC patients treated with RC or NMIBC patients treated with TURBT.

### 3.7. Results regarding blood type rhesus factor

Based on 4 studies,^[22,24,26,27]^ the outcomes of pooled results did not demonstrate an influence of positive RH (Ref: negative) on disease recurrence-free OS, CSS, RFS, recurrence, CSM and OM of the BC patients treated with RC.

## 4. Discussion

There are many studies regarding ABO blood group and rhesus factor which reported different impact on different types of cancers such as breast cancer^[29]^ and pancreatic cancer^[30]^. Our research summarized that the prognosis of ABO blood group and rhesus factor on outcomes in patients with BC and demonstrated that ABO blood group might have impact on BC who underwent RC while rhesus factor has no impact on BC. In the present study, we found that blood type A may be relevant to NMIBC patients treated with TURBT; in NMIBC patients treated with TURBT, blood type B patients have a rather lower incidence of disease recurrence and progression; although blood type O and non-O blood type have not been found to be related to disease recurrence, blood type O and non-O blood type are still associated with CSM in multivariable analyses. Besides, comparing with blood type O, blood type B doesn’t have statistical significance for BC patients accepted RC treatment. The same results were shown for blood type AB NMIBC patients treated with RC, either.

The ABO blood group antigens and the rhesus factor could impact the cancer progression and survival via various mechanisms. Some clinical trials concluded that an association between gene polymorphisms. ABO gene is associated with levels of tumor necrosis factor-alpha,^[31]^ soluble intercellular adhesion molecule (ICAM), E-selectin and P-selectin.^[32,33]^ All these molecules could be important mediators of immune cell recruitment. Hence, the role of genetic factors in the development of cancer can’t be ignored. And some studies concluded that cancers which most frequently metastasize are associated with the level of inflammation molecules. Therefore, these molecules may provide a biological basis for the postulated influence of ABO blood types on OS of patients, by directly linking ABO blood group, tumor initiation and tumor spread. What’s more, human epithelial cells of type O individuals expressed significantly more *Helicobacter pylori* and had more inflammatory responses to *H. pylori* than epithelial cells of persons with other blood types.^[34,35]^ Other experimental studies have provided strong biological explanations for the higher prevalence of some diseases among individuals with blood type O than type A.^[36]^ For patients with BC, ABO antigens are changed or lost in UCB cells.^[37,38]^ Abnormal glycosylation of cell surface proteins may modulate epithelial-mesenchymal transition, which is a crucial step in cancer development and progression.^[39]^ Finally, the A and B blood group antigens are associated with an increased risk of thromboembolism, thus affecting OS.^[40]^ In the majority of cancer entities, conflicting results regarding the impact of the ABO blood group antigen expression on survival have been reported.^[14,31,41]^ Liu et al^[42]^ reported that the relationship between ABO blood group and Von Willebrand factor (VWF), factor VIII and ADAMTS-13 levels in lung cancer patients and the results showed that compared with type O blood, the plasma VWF level and FVII activity in non-O blood were significantly increased, and the ADAMTS-13 level in control group was significantly decreased. The increase of VWF and the decrease of ADAMTS-13 promote the invasion and metastasis of lung cancer. A study of 136 patients with metastatic renal cell carcinoma who received tyrosine kinase inhibitors (TKIs) as the first-line treatment showed that type O blood was significantly correlated with the increase in the number of disease sites.^[43]^ Srinivas et al^[44]^ analyzed the clinical data of 141 patients with BC and found that patients with type A blood had lower tumor grade and lower mortality, while patients with type O blood had higher tumor grade and higher mortality. Orihuela et al^[45]^ also reached a similar conclusion that the clinical data of 494 patients with superficial BC showed that patients with blood group O had a significantly higher risk of advanced disease than other patients. Although Llopis et al^[46]^ and Raitanen et al^[47]^ also observed that the oncological behavior of patients with type O blood was higher than that of patients with type A blood, with higher tumor grade and recurrence rate, this difference was not statistically significant.

The effects of ABO blood type on prognosis of different cancers remain controversial. Among previous studies that have investigated the association of the ABO blood type and pancreatic cancer, they mainly showed no association.^[15,48–50]^ Akin et al^[29]^ investigated whether ABO blood group and rhesus factor have connections with the risk of getting breast cancer, and this research including 3944 patients diagnosed with breast cancer in Turkey showed that patients with breast cancer have the same distribution of ABO blood group as the healthy ones, but what could be anticipated is that patients with blood type O are more likely having a better prognosis. Another single-center study in China demonstrated that there exist association between ABO blood group and the risk of incidence of gastric cancer. Generally, the related indexes of ABO blood group test, as cheap indexes in clinical laboratory, have certain significance in judging the prognosis of patients with gastric cancer, but the specific mechanism is still unclear. Thus, further investigations are needed to verify those findings. Interestingly, a meta-analysis^[51]^ also found that gastric cancer with blood type A patients are more prone to *H. pylori* infection than other ABO blood type individuals. In all, ABO blood types on prognosis of different cancers are still controversial. Considering our systematic review, some results are also controversial for NMIBC treated with TURBT. Cheng^[19]^ reported that patients with blood type O have higher recurrence and progression rate than type A while Wang^[21]^ and He^[20]^ reported that patients with blood type O have lower recurrence and progression rate than type A.

The current study has a few limitations. First, because RCTs and observational studies were combined in the analysis, the results should be treated with caution. Second, all of the included studies were conducted in Chinese populations. Therefore, physicians around the world should interpret our results with caution when applying them in clinical practice. Considering the ideal biomarker should allow identification of patients at risk of a certain outcome with acceptable cost.^[52]^ Despite these methodological limitations, we provide evidence that some merit as markers of BC prognosis.

## 5. Conclusion

Our study confirms that the ABO blood group antigens are associated with the prognosis of BC patients. We noted a particular association of blood type for prognosis of patients with BC and ABO blood group antigen expression can be suitable biomarkers for BC. However, the association of the ABO blood group antigens on BC patients is not totally verified, although they are available in every BC patient undergoing surgery. We also found that rhesus factor has no impact on prognosis of BC patients.

## Acknowledgments

The authors thank Xin He, from the Qinghai University, for reviewing this paper and his helpful comments.

## Author contributions

**Conceptualization:** Haiming Yang and Jingxin Yan.

**Data curation:** Haiming Yang and Jingxin Yan.

**Formal analysis:** Haiming Yang and Jingxin Yan.

**Investigation:** Haiming Yang and Jingxin Yan.

**Methodology:** Haiming Yang and Jingxin Yan.

**Project administration:** Jingxin Yan.

**Resources:** Haiming Yang and Jingxin Yan.

**Software:** Haiming Yang and Jingxin Yan.

**Supervision:** Jingxin Yan.

**Validation:** Jingxin Yan.

**Visualization:** Jingxin Yan.

**Writing – original draft:** Haiming Yang and Jingxin Yan.

**Writing – review & editing:** Haiming Yang and Jingxin Yan.
